# Supplementary material for: GRAND: a database of gene regulatory network models across human conditions
Source: Nucleic Acids Res. 2021 Sep 11;50(D1):D610–21. doi: 10.1093/nar/gkab778 (PMC8728257; doi:10.1093/nar/gkab778)
Supplement: gkab778_Supplemental_File [file gkab778_supplemental_file.pdf]

Supplementary information

GRAND: A database of gene regulatory network models across human conditions

Ben Guebila, Lopes-Ramos et al.

Outline

I. **Supplementary methods**
 1. Database content
 2. Browsing phenotypic information
 3. CLUEreg: Drug repurposing analysis using gene regulatory networks

II. **Supplementary figures**
 1. Figure S1 - Screenshot of small molecule, cell line, cancer types, and tissues summary plots in GRAND.
 2. Figure S2 - Summary statistics of the cancer resource and the tissues resource.
 3. Figure S3 - The small molecule resource integrates drug characteristics with cell line phenotypic information.

III. **Supplementary tables**
 1. Table S1 - Database content by condition and regulation modality.

I. Supplementary methods

1. Database content

GRAND is a large-scale, multi-study catalog of GRNs that provides regulatory models for perturbed and unperturbed human cell lines, as well as normal and cancer tissues. These models were generated using data from large repositories including GTEx, TCGA, CCLE, and the Connectivity Map, as well as selected studies from GEO. The GRNs in GRAND are classified into four large groups—small molecule screens, cancer tissues, normal tissues, and cell lines. GRAND allows users to browse, visualize, analyze, and download these GRNs either through the web interface or programmatically through GRAND's API. GRAND also allows network-based queries to identify small molecule candidate drugs that can potentially correct altered regulatory processes in disease states and users can upload their own networks to run the collection of tools in GRAND.

The GRNs hosted in GRAND, including the inference pipeline to generate each network, are accessible through an interactive web interface as well as through a well-defined application program interface (API). A network visualization module allows the users to query and plot subnetworks of interest based on several selection parameters as well as to compute the corresponding targeting scores. To support analysis of the collection of networks, we developed two web server applications that allow users to query the GRAND database. The first allows users to perform functional enrichment analysis on a set of TFs ranked by targeting score. The second utility is similar to Connectivity Map analysis (1), but uses network features instead of expression to identify candidate drugs and drug combinations that could be used to reverse or alter regulatory patterns in a particular disease state. Finally, users can upload their own networks for visualization and analysis in GRAND. We demonstrated the utility of GRAND by presenting an example in which we compare GRNs between colon cancer and normal colon tissue to identify the genes that are differentially targeted by key regulatory TFs. We then use these to identify an investigational drug that may have a specific effect in colon cancer (Figure 4).

2. Browsing phenotypic information

Each network page has a table of phenotypic information about the samples used for network reconstruction process. For aggregate networks, this table was intended to give information about the samples and classify them by variables such as sex, age, ethnicity, and survival. For single-sample networks, the phenotypic information page allows the user to visualize and download the sample-specific network. To facilitate the selection of networks, phenotypic variables are classified into continuous variables, such as height and age, and categorical variables, such as sex and ethnicity. Continuous variables are plotted as scatter plots at the top of the network page. Clicking on an individual sample within the plot links to the network visualization page. When continuous variables are missing, we display additional information about the data such as the number of differentially targeted and expressed genes and TFs in each cell line and drug sample, as well as the top ten enriched GO terms for differential TF and gene targeting in cancer. Categorical variables are plotted using pie charts in the phenotypic information page. Clicking on each category within individual pie charts filters the phenotypic information page by the selected phenotype.

3. CLUEreg: Drug repurposing analysis using gene regulatory network

To build CLUEreg, we extended the small molecule resource in GRAND to all the approved and experimental drugs profiled in the Connectivity Map, consisting of 19,791 total small molecules. Because each small molecule is administered to multiple cell lines using a variety of doses and

sampling times, we used PANDA to build an aggregate GRN for each drug, totaling 19,791 GRNs. For each drug-specific GRN, we constructed a "targeting score" for each gene as the sum of inbound edge weights. For each TF, we calculated a targeting score as the sum of outbound edge weights. The targeting score of all drug-specific GRNs are assembled into a gene-by-drug or TF-by-drug targeting matrix. We then reduce the complexity of these matrices to the set of "differentially targeted/targeting" genes or TFs by comparing the targeting weight to the distribution of weights within the matrix and selecting as differentially targeted/targeting those genes/TFs that have targeting scores that deviate with more than two standard deviations from the mean.

To use CLUEreg, users provide two lists, one consisting of genes (or TFs) with increased targeting and the second consisting of genes (or TFs) with decreased targeting in the disease of interest. These are compared to the library of 19,791 drug-specific GRNs to identify small molecule drug treatments that likely reverse the targeting score of the gene/TF in the original input GRN. For a given input of a differentially targeted gene (or TF) list, CLUEreg computes two measures of agreement with the effect of each drug (Figure 3).

The first is the cosine similarity comparing the differentially targeted gene lists in a user's input query and a specific drug as described in Duan et al. (2). A cosine similarity equal to -1 indicates that the drug has a regulatory pattern that is the reverse of the input list, suggesting that the drug is a candidate for reversing the differential regulation induced by the disease state under investigation. In contrast, a cosine similarity equal to 1, indicates that the small molecule exacerbates the input list, as it aligns perfectly with its direction and sense.

The second measure computed by CLUEreg is the overlap score (2) between the input list and the differentially targeted genes (or TFs) for each drug, defined as:

$$Overlap = |Input\_Genes\_Up \cap Drug\_Genes\_Down\,| + |Input\_Genes\_Down \cap Drug\_Genes\_Up\,| \\ -|(Input\_Genes\_Up \cap Drugs\_Genes\_Up\,| - |(Input\_Genes\_Down\ \cap Drugs\_Genes\_Down\,)|,$$

Where $Input\_Genes\_Up$ refers to the list of user-given high-targeted genes, $Drug\_Genes\_Down$ is the list of low-targeted genes in a given drug network, $Input\_Genes\_Down$ is the the list of user-given low-targeted genes, and $Drug\_Genes\_Up$ is the list of high-targeted genes in a given drug network. A positive overlap score between a query targeting list and a drug targeting list suggests that the drug reverses the input, while a negative overlap score suggests that the drug and the input have similar regulatory effects. In developing the application, we have found that both metrics provide highly consistent rankings of candidate drugs.

CLUEreg computes a p-value for each drug candidate by resampling 10,000 random inputs of varying lengths as a null distribution. In addition, q-values are provided as corrected p-values using the Benjamini-Hochberg procedure (3). There are several drug classes that can induce profound changes on transcription and often produce false positives in connectivity analysis. An example of such drugs are Histone Deacetylase (HDAC) inhibitors. To control for these effects, we computed a tau-value as described in the Connectivity Map (1). First, we computed the cosine similarity of each drug in CLUEreg against all other drugs to generate a cosine similarity distribution. Then to generate the tau-value, we rank the cosine similarity between the input query and a given drug within the precomputed distribution of all drugs. Tau varies between 0 and 1 and represents the fraction of drugs in the database that have a stronger connectivity. Low tau-values indicate specific activities, while large tau values indicate compounds with promiscuous effects.

We also implemented drug combinations in CLUEreg as described in Duan et al. (2) by ranking pairs of drugs within the top 20 hits. Drug pairs are ranked by their cosine similarity such that an optimal pair has a cosine similarity of 0, which indicates activity on orthogonal gene/TF vectors.

Therefore, the optimal drug combination has compounds that optimally reverse the input regulatory profiles while acting on different target genes and pathways.

II. Supplementary figures

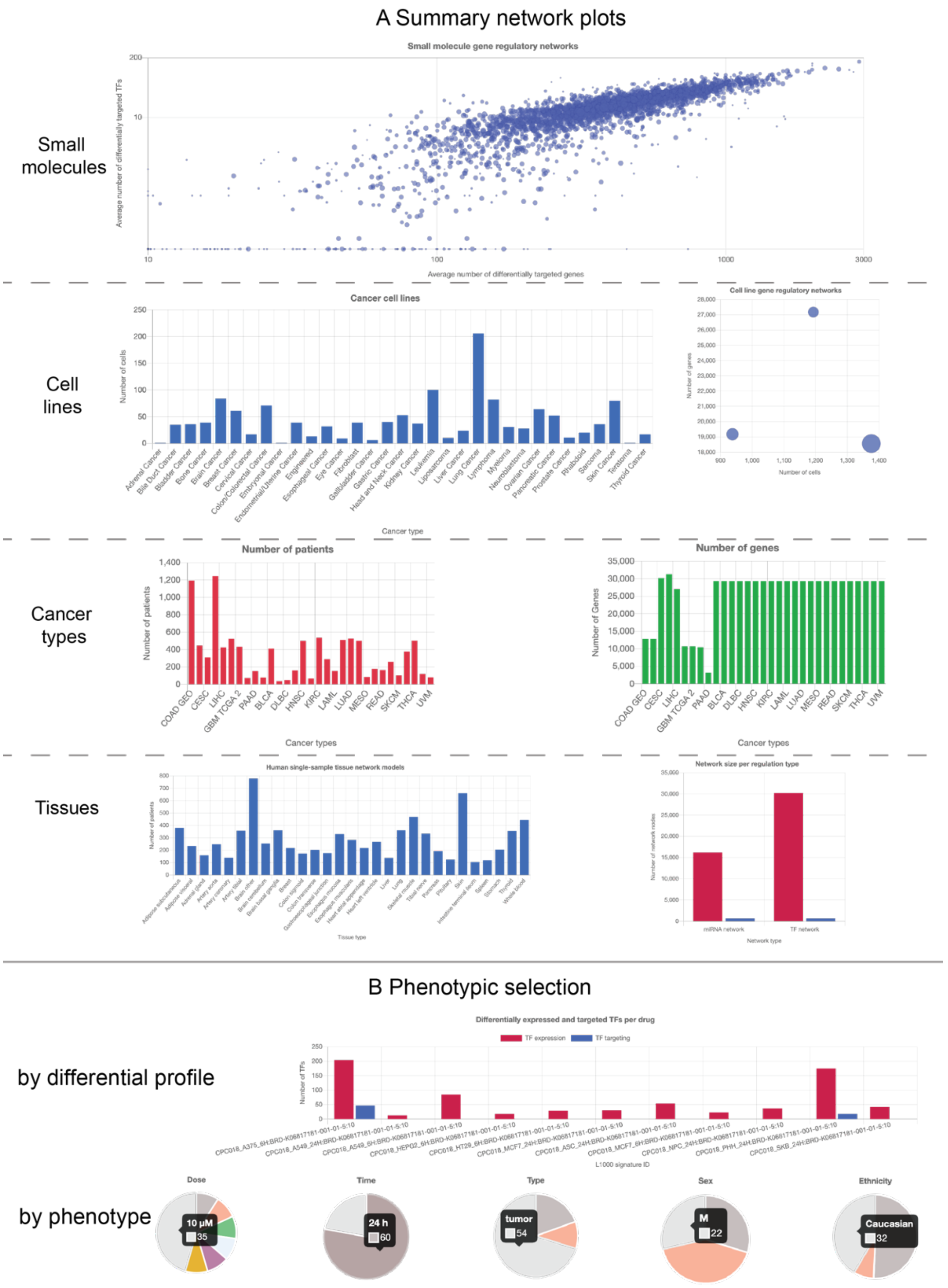

Figure S1 - Screenshot of small molecule, cell line, cancer types, and tissues summary plots in GRAND. A. The main page for each resource displays a summary interactive plot for the catalog of networks. For small molecules, a bubble plot for each compound leads to the targeting scores across doses, cell lines and sampling times. Cell line, tissue, and cancer TF and miRNA networks are organized by tissue of origin. **B.** A sample-specific network can be selected

interactively by differential expression or targeting score of TFs and genes or by phenotypic variables such as donor age, sex, and ethnicity.

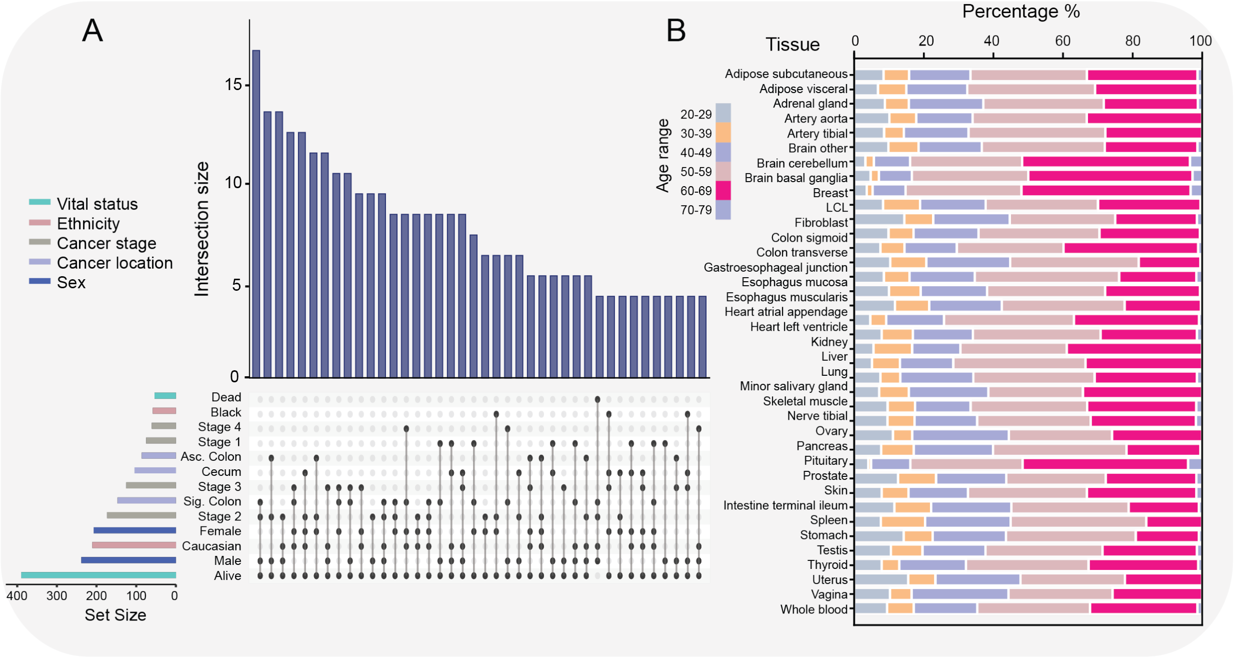

Figure S2 - Summary statistics of the cancer resource and the tissues resource. A. UpSet plot of the set intersection size of the most important clinical attributes in the cancer resource using colon cancer as an example. The plot represents the intersection between different groups of clinical attributes, for example the first group has 16 patients that belong to the groups “alive, “ “stage 2” cancer, and with the cancer located in the “sigmoid colon.“ **B.** Age distribution of the subjects from GTEx included in the tissues resource**.**

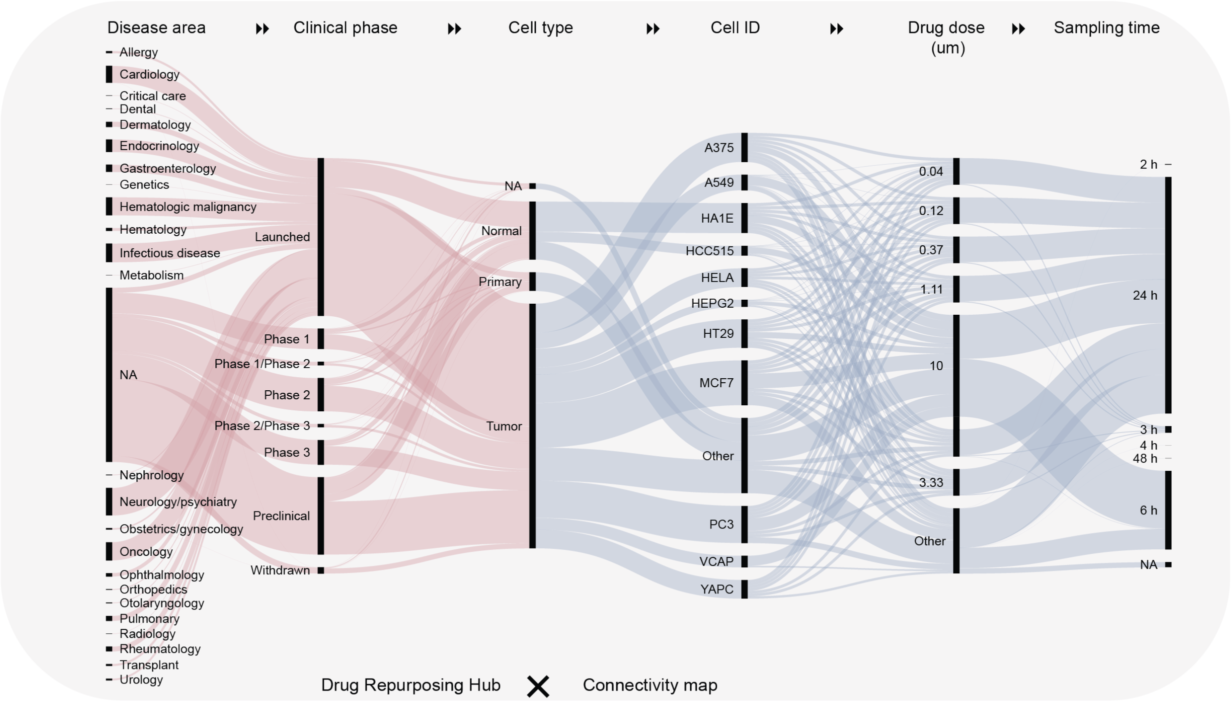

Figure S3 - The small molecule resource integrates drug characteristics with cell line phenotypic information. The resource combines information from the Connectivity Map and the Drug Repurposing Hub (DRH) for more than 173,013 samples. Each sample is represented by an edge in the diagram.

III. Supplementary tables

Table S1 - Database content by condition and regulation modality. PAAD: Pancreatic adenocarcinoma, GBM: Glioblastoma multiforme, COAD: Colon adenocarcinoma.

Resource	Types	Data set	Regulation	Number of GRNs	Network type	Reference
**Cell lines**	LCL, Fibroblast	GTEx	TF	2	Aggregate	(4)
**Cell lines**	35 tissues of origin	CCLE	TF	1,376	Single-sample	This paper
**Cell lines**	35 tissues of origin	CCLE	miRNA	1	Aggregate	This paper
**Tissues**	36 tissue types	GTEx	TF	36	Aggregate	(5)
**Tissues**	36 tissue types	GTEx	miRNA	36	Aggregate	(6)
**Tissues**	29 tissue types	GTEx	TF	8,279	Single-sample	(7)
**Cancer**	PAAD	TCGA	TF	150	Single-sample	(8)
**Cancer**	GBM	TCGA/GGN	TF	1,023	Single-sample	(9)
**Cancer**	COAD	TCGA/GEO	TF	1,638	Single-sample	(10)
**Cancer**	All	TCGA	TF	22	Aggregate	This paper
**Small molecules**	2,858 labeled drugs	CLUE/DRH	TF	173,013	Targeting scores	This paper

References

1. Subramanian, A., Narayan, R., Corsello, S.M., Peck, D.D., Natoli, T.E., Lu, X., Gould, J., Davis, J.F., Tubelli, A.A. and Asiedu, J.K. (2017) A next generation connectivity map: L1000 platform and the first 1,000,000 profiles. *Cell*, **171**, 1437-1452. e1417.
2. Duan, Q., Reid, S.P., Clark, N.R., Wang, Z., Fernandez, N.F., Rouillard, A.D., Readhead, B., Tritsch, S.R., Hodos, R. and Hafner, M. (2016) L1000CDS 2: LINCS L1000 characteristic direction signatures search engine. *NPJ systems biology and applications*, **2**, 1-12.
3. Benjamini, Y. and Hochberg, Y. (1995) Controlling the false discovery rate: a practical and powerful approach to multiple testing. *Journal of the Royal statistical society: series B (Methodological)*, **57**, 289-300.
4. Lopes-Ramos, C.M., Paulson, J.N., Chen, C.-Y., Kuijjer, M.L., Fagny, M., Platig, J., Sonawane, A.R., DeMeo, D.L., Quackenbush, J. and Glass, K. (2017) Regulatory network changes between cell lines and their tissues of origin. *BMC genomics*, **18**, 1-13.
5. Sonawane, A.R., Platig, J., Fagny, M., Chen, C.-Y., Paulson, J.N., Lopes-Ramos, C.M., DeMeo, D.L., Quackenbush, J., Glass, K. and Kuijjer, M.L. (2017) Understanding tissue-specific gene regulation. *Cell reports*, **21**, 1077-1088.
6. Kuijjer, M.L., Fagny, M., Marin, A., Quackenbush, J. and Glass, K. (2020) PUMA: PANDA Using MicroRNA Associations. *Bioinformatics*, **36**, 4765-4773.
7. Lopes-Ramos, C.M., Chen, C.-Y., Kuijjer, M.L., Paulson, J.N., Sonawane, A.R., Fagny, M., Platig, J., Glass, K., Quackenbush, J. and DeMeo, D.L. (2020) Sex differences in gene expression and regulatory networks across 29 human tissues. *Cell reports*, **31**, 107795.
8. Weighill, D., Guebila, M.B., Glass, K., Platig, J., Yeh, J.J. and Quackenbush, J. (2021) Gene targeting in disease networks. *arXiv preprint arXiv:2101.03985*.
9. Lopes-Ramos, C.M., Belova, T., Brunner, T., Quackenbush, J. and Kuijjer, M.L. (2021) Regulation of PD1 signaling is associated with prognosis in glioblastoma multiforme. *bioRxiv*.
10. Lopes-Ramos, C.M., Kuijjer, M.L., Ogino, S., Fuchs, C.S., DeMeo, D.L., Glass, K. and Quackenbush, J. (2018) Gene regulatory network analysis identifies sex-linked differences in colon cancer drug metabolism. *Cancer research*, **78**, 5538-5547.
